# Predictive Performance of Serum β-hCG MoM Levels for Preeclampsia Screening: A Meta-Analysis

**DOI:** 10.3389/fendo.2021.619530

**Published:** 2021-06-10

**Authors:** Xiao Zhang, Zhao Huangfu, Fangxin Shi, Zhen Xiao

**Affiliations:** ^1^ Department of Obstetrics and Gynecology, First Affiliated Hospital of Dalian Medical University, Dalian, China; ^2^ Department of Urology, The Second Affiliated Hospital of Dalian Medical University, Dalian, China; ^3^ Institute of High Altitude Medicine, People’s Hospital of Naqu Affiliated to Dalian Medical University, Naqu, China

**Keywords:** preeclampsia, β-hCG MoM levels, prediction, early second trimester, meta-analysis

## Abstract

**Objective:**

The aim of the present study was to investigate the predictive value of using the multiple of the median (MoM) of β-human chorionic gonadotropin (β-hCG) levels in patients with preeclampsia (PE) and healthy pregnant women.

**Methods:**

Electronic databases including PubMed, EBSCO, Ovid, Web of Science, China National Knowledge Infrastructure (CNKI), SinoMed, Wangfang and the Weipu Journal were searched up to May 31, 2020. Two reviewers independently selected the articles and extracted data on study characteristics, quality and results. A random-effects model was employed, and standardized mean difference and 95% confidence intervals were calculated. Twenty-one case-control studies were analyzed in the present meta-analysis, including a total of 2,266 cases and 25,872 healthy controls.

**Results:**

Women who were diagnosed with PE were found to have higher early second-trimester levels of serum β-hCG MoM compared with healthy controls, although the levels in the first trimester were not significantly different. Ethnicity subgroup analysis demonstrated that the MoM of β-hCG serum levels was significantly higher in PE patients in both Asian and Caucasian populations during the early second trimester.

**Conclusion:**

The MoM of β-hCG serum levels was found to be a valuable clinical indicator for predicting PE in the early second trimester, but had little predictive value in the first trimester. However, further assessment of the predictive capacity of β-hCG within larger, diverse populations is required.

## Introduction

Preeclampsia (PE) is an idiopathic disease of pregnancy that may lead to multi-organ damage, and it is a multi-systemic disorder that is associated with poor early placentation and is characterized by new-onset hypertension and proteinuria after 20 weeks of gestation, with a significant impact on several organ systems, including renal and hepatic insufficiency, neurological complications, hematological complications, or evidence of uteroplacental dysfunction (1–4). PE is the second leading cause of maternal mortality on a global scale, and it is the main cause of pregnancy-related hospitalization. The prevalence of PE globally is 2-10%, while the prevalence is higher (4-18%) in developing countries (5, 6). To date, prenatal control for pregnant women and active termination of pregnancy in patients suffering from severe PE are the only effective methods for decreasing morbidity and mortality of pregnant women and their fetuses.

Although the factors affecting PE remain unclear, most studies suggest that the main pathogenic mechanism maybe inadequate trophoblast invasion into maternal spiral arteries, resulting in decreased placental blood flow, trophoblast apoptosis and pro-inflammatory cytokine production (7). Recent research using maternal blood and placentas from PE patients also indicates that the pathophysiological origin of PE maybe traced back to the placenta. Therefore, it would be of great value to accurately predict PE by identifying early proteomic biomarkers of placental dysfunction in order to apply timely interventions to reduce the prevalence of PE. To date, several biochemical markers of placental dysfunction have been used to evaluate the predictive factors of PE before the appearance of clinical symptoms. Some studies indicated that β-human chorionic gonadotropin (β-hCG) is involved in PE, and β-hCG has been recommended as a serum maker for screening PE at 8-14 weeks of gestation (8, 9).

β-hCG is a glycoprotein hormone produced by trophoblast cells, which is often used to diagnose pregnancy, ectopic pregnancy and hydatidiform mole. Trophoblasts are specialized cells of the placenta that play an important role in the exchange of gases and nutrients between them other and the fetus and are involved in blastocyst attachment, placental implantation and placental vasculature (10). During normal pregnancy, the concentration of β-hCG peaks between 10 and 12 weeks, and then gradually decreases. Abnormal placental formation/function may induce changes in serum β-hCG levels (11).

Several studies have reported an association between the reduction of β-hCG MoM levels in the first trimester and the development of PE (12); however, some findings are inconsistent (13, 14). Some studies suggested that β-hCG MoM levels were only increased in patients with severe PE, while there was no significant change in patients with mild PE (13). Although some scholars conducted a meta-analysis of the predictive value of serum β-hCG MoM levels indicating that β-hCG MoM levels were significantly increased in women with PE compared with healthy pregnant women, that study did not analyze the effect of the timing of serum β-hCG detection on its predictive value for PE (9).Subsequently, numerous studies analyzed the association between serum β-hCG MoM levels and PE, and a number of articles indicated that there was no statistically significant association between serum β-hCG MoM levels and PE (15–18). Therefore, a meta-analysis was conducted hereinto evaluate and verify the predictive value of MoM of β-hCG serum levels in PE.

## Materials and Methods

### Literature Search

The electronic databases PubMed, EBSCO, Ovid, Web of Science, China National Knowledge Infrastructure (CNKI), SinoMed, Wangfang and the Weipu Journal were searched (last search: May 31, 2020) for published studies relevant to our topic of interest, without restriction of language or data collection. Search terms were combined with MeSH terms (‘Chorionic Gonadotropin, beta Subunit, Human’ or ‘Chorionic Gonadotropin’) and (Pre-eclampsia’) with different search terms/key words in all fields (‘β-hCG’ or ‘β-hcg’ or ‘beta-hcg’ or ‘human chorionic gonadotropin’ or ‘hcg’) to retrieve the relevant articles from the databases. Manual searches of cross-references were also conducted to screen for other eligible studies. The meta-analysis was registered in PROSPERO (CRD42020190736).

### Study Selection

The first stage of study selection involved scrutinizing the database by 2 independent reviewers (Xiao Zhang and Zhao Huangfu) to identify articles from the title and abstract review based on keywords for β-hCG MoM and PE. In the second stage, the titles and abstracts of all the identified citations were also independently screened to identify studies that fulfilled the inclusion criteria by the same 2 reviewers to confirm study eligibility. It was discussed with senior reviewer (Fangxin Shi) if any disagreement occurred. The criteria for inclusion were as follows: i) Published studies investigating the association between β-hCG MoM and PE; ii) prospective, retrospective or nested case-control design; iii) diagnosis of PE confirmed by the American College of Obstetricians and Gynecologists (ACOG) [hypertension (systolic blood pressure≥140 mm Hg and/or diastolic blood pressure≥90 mm Hg, on two separate measurements conducted at least 44 h apart) and proteinuria (≥0.3 g/day urine collection and/o≥1+ on dipstick testing) after 20 weeks of gestation] and the guidelines of the International Society for the Study of Hypertension in Pregnancy (ISSHP) [systolic BP≥140 mmHg or diastolic BP≥90 mmHg on two measurements at least 4 h apart in previously normotensive women after 20 weeks of gestation, and proteinuria of 300 mg or more in 24 h] (19, 20); iv) original data provided; v) studies including singleton pregnancies only. The criteria for exclusion were abstracts, editorials, narrative reviews, case reports, letters to the editor, and meta-analyses or other types of articles that did not include primary study results. Additionally, duplicate publications or studies with overlapping data were not considered.

### Data Extraction and Quality Assessment

Two independent investigators (Xiao Zhang and Zhao Huangfu) extracted a range of data from each study using a standardized data-collecting form: First author’s name, year of publication, country, ethnicity, population evaluated, study design, definition and number of cases and controls, maternal age range or mean age, systolic blood pressure, diastolic blood pressure or mean arterial pressure, demographic variables, disease type, protein expression levels and P-values between cases and controls. The authors were contacted by email in cases of missing information. The methodological quality of the included trials was evaluated based on the Newcastle-Ottawa Scale (NOS) by the same 2 investigators respectively. Disagreements were resolved through discussion, or by consulting a third investigator (Fangxin Shi).

### Statistical Analysis

All the continuous data (serum β-hCG MoM levels) was presented as mean ± standard deviation (SD). Serum concentration given as median and range were converted into mean and SD using the formula reported by Luo et al. (21), Wan et al. (22) and Shi et al. (23). The strength of the association between serum β-hCG MoM levels and PE was estimated by standardized mean differences (SMDs) and 95% confidence intervals (95% CIs) calculated by the Z-test. Between-study heterogeneity was evaluated by the Cochran’s Q statistic (*P*<0.05 was considered significant) and I^2^ tests (24). To calculate the pooled SMDs, fixed/random-effects models were used; a random-effects model was applied when there was evidence of significant heterogeneity (*P*<0.05 or *I* ^2^>50%), and odds ratios were pooled based on the fixed-effects model (25, 26). If there was significant heterogeneity, subgroup analysis based on ethnicity and sample size was performed to identify potential explanatory variables for the differences in serum β-hCG MoM levels between cases and controls. Source of heterogeneity estimation was performed by univariate and multivariate meta-regression analyses, and further verification was conducted using Monte Carlo simulation (27–29). In addition, sensitivity analyses were performed to assess the effect of each single study and the stability of the meta-analysis results by sequential removal of individual studies. Furthermore, the effect of publication bias was detected by funnel plot, Galbraith plot and Egger’s linear regression test (*P*<0.05 was considered significant) (30, 31). Statistical analyses were conducted with Stata statistical software, version 15.1 (Stata Corporation, College Station, Texas, USA).

## Results

### Selection of Eligible Studies

**Figure 1:**
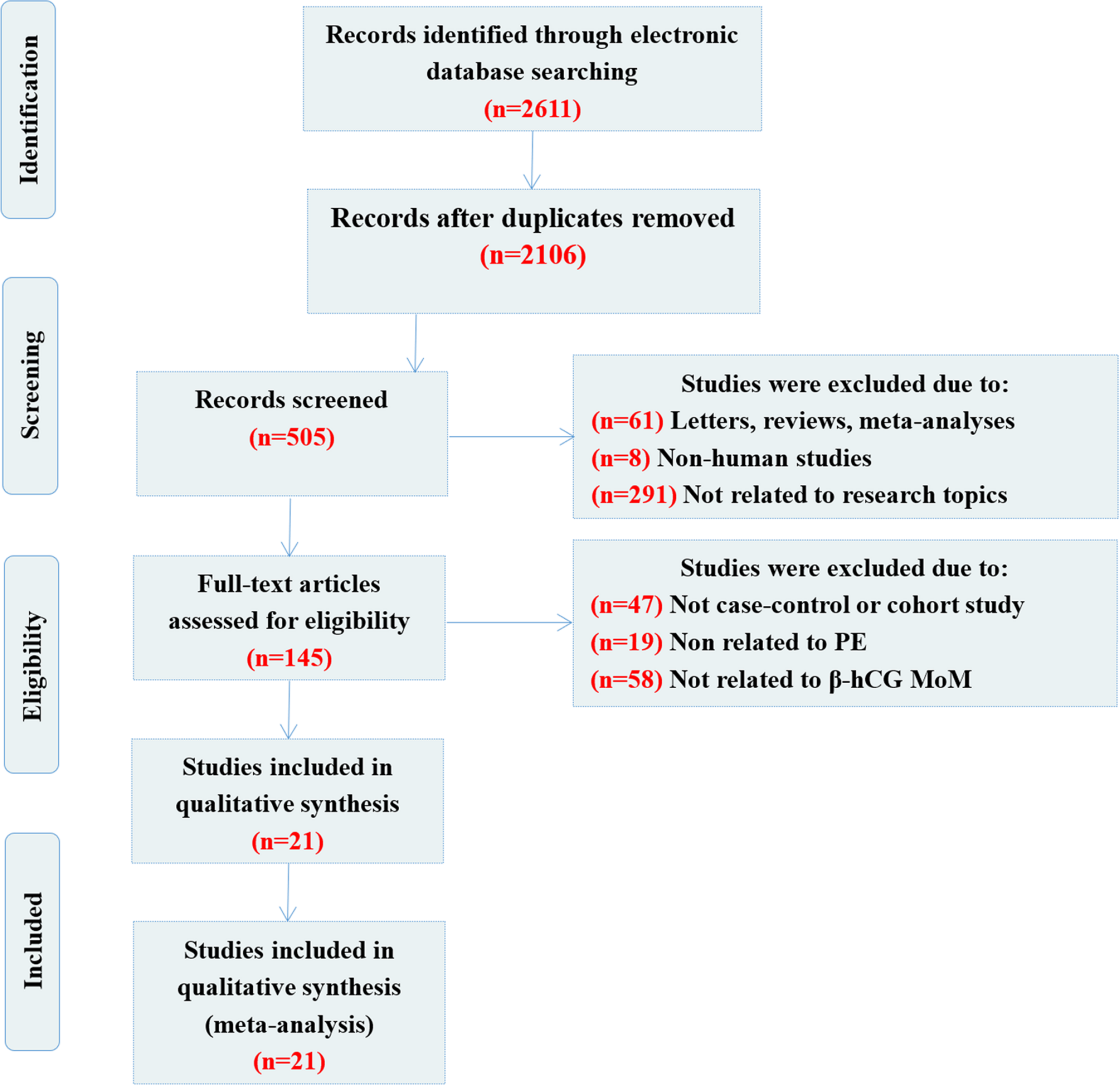
Flow chart showing the results of the search strategy.

A flow chart showing the detailed study inclusion and exclusion process is presented in **Figure 1**. The primary search yielded 2,611 articles (including a manual search, n=2), 2,106 of which were repetitive publications and were excluded. A total of 360 articles were removed as they were reviews or meta-analyses (n=61), non-human studies (n=8) or unrelated to the topic (n=291). After further evaluation, 124 studies were removed following a more detailed full-text assessment, including 47 studies that were not cohort or case-control studies, 19 studies that were unrelated to PE and 58 that were unrelated to β-hCG MoM. Finally, 21 case-control publications were selected for inclusion in this meta-analysis (12–18, 32–45). The enrolled studies were of moderate to high quality, and the NOS assessments for each included study are summarized in **Table 1**.

**Table 1:** Characteristics of the included studies.

Reference(first author)	Year	Country	Ethnicity	Detection method	Detection period, weeks		Number			Maternal age, years mean ± standard deviation or median (interquartile ranges)		*P* value	NOS score
					case	control	size	sam-ple	case/control	case	control		
Long W	2018	China	Asian	TRFIA	15-20	15-20	large	1369	198/1171	27.7 ± 3.6	26.6 ± 3.5	0.09	6
Papastefanou I	2018	Germany	Caucasians	ELISA	11-14	11-14	small	277	12/265	31.9 ± 3.7	31 ± 4.2	0.958	7
Yu N	2017	China	Asian	TRFIA	11-13^+6^	11-13^+6^	large	662	462/200	30.2 ± 4.6	28.6 ± 2.3	0.536	7
Kim SY	2016	Korea	Asian	TRFIA	15-20	15-20	small	118	34/84	34.1(32.7-36.0)	33.0(31.0-35.3)	>0.05	6
Zheng QZ	2016	China	Asian	ELISA	15-20	15-20	small	259	63/196	31.8 ± 4.3	31.5 ± 7.5	0.013	6
Crovetto F	2015	Spain	Caucasians	DELFIA	10.6 ± 3.9	10.6 ± 3.9	small	340	40/300	33.9 ± 6.4	32.7 ± 4.7	<0.05	7
Crovetto F	2014	Spain	Caucasians	DELFIA	10.1(9.1-10.6)	10.2(9-10.6)	large	9462	303/9159	32.3 ± 6.1	31.7 ± 5.3	<0.001	8
Karahasanovic A	2014	Denmark	Caucasians	ELISA	11.1(8-14)	11(8-11)	small	249	161/88	30.9(18.8-41.2)	28.1(21.1-40.7)	<0.05	7
Ozdamar O	2014	Turkey	Caucasians	UA	12.4 ± 0.6	12.4 ± 0.7	small	240	60/180	29.3 ± 5.7	28.1 ± 4.3	0.882	6
Teixeira C	2014	Portugal	Caucasians	DELFIA	9-13^+6^	9-13^+6^	large	4799	140/4659	31.0(27.7-33.6)	29.9(25.8-33.0)	>0.05	6
Lai J	2013	UK	Caucasians	DELFIA	11-13^+6^	11-13^+6^	small	300	50/250	29.8(24.2-33.8)	31.2(27.6-34.9)	>0.05	7
Suri S	2013	UK	Caucasians	DELFIA	11-14	11-14	small	56	14/42	NR	NR	NS	7
Kuc S	2013	Netherlands	Caucasians	DELFIA	12.0 ± 1.2	12.5 ± 0.7	large	667	167/500	33.2 ± 4.8	32.6 ± 3.7	NS	6
Wortelboer EJ	2010	Netherlands	Caucasians	DELFIA	8-13^+6^	8-13^+6^	large	568	88/480	34.6(31.0-37.3)	36.0(33.3-38.2)	0.93	7
Staboulidou I	2009	UK	Caucasians	NR	11-13	11-13	large	2029	165/1864	31.9 ± 7.0	32.0 ± 5.9	NS	6
Spencer K	2006	UK	Caucasians	ELISA	11-13^+6^	11-13^+6^	small	168	24/144	NR	NR	0.003	7
Spencer K	2005	UK	Caucasians	ELISA	11-13^+6^	11-13^+6^	large	4063	64/3999	29(17-44)	30(16-47)	0.266	6
Tsai MS	2002	Taiwan	Asian	ELISA	10-13	10-13	large	711	46/665	30(20-43)	NR	NS	6
Lee LC	2000	Taiwan	Asian	MEIA	15-20	15-20	large	1052	95/957	30.0 ± 4.7	28.6 ± 4.1	<0.001	6
Raty R	1999	Finland	Caucasians	TRFIA	16.4 ± 1.5	15.9 ± 1.2	small	343	10/282	26.2 ± 3.0	29.7 ± 3.5	>0.05	7
Luckas M	1998	UK	Caucasians	RIA	15-18	15-18	small	406	19/387	22 ± 5.4	25.1 ± 5.6	0.03	6

DELFIA, Dissociation-enhanced lanthanide fluoroimmunoassay; UA, ultrasonographic assessment; ELISA, enzyme-linked immunosorbent assay; MEIA, microparticle enzyme immunoassay; TRFIA, time-resolved fluorescence immunoassay; RIA,radioimmunoassay; TSI, two-site immunometric assay; CLIA, chemiluminescence immunoassay; NR, not reported; NS, no significance.

### Baseline Information of the Included Studies

The 21 selected studies included a total of 28,138 subjects, with 2,266 cases and 25,872 healthy controls. The sample size ranged between 56 and 9,462. Of these studies, 15 were in Caucasian populations and 6 studies were performed in Asian populations. β-hCG was detected in the early second trimester in 6 studies and in the first trimester in the remaining studies; 10 studies were large-sample studies and the remaining 11 were small-sample studies. A total of 6 different methods were used to measure serum β-hCG levels in the included studies: Dissociation-enhanced lanthanide fluoroimmunoassay was used in 7 studies, time-resolved fluorescence immunoassay was used in 4 studies, enzyme-linked immunosorbent assay was used in 6 studies, and the remaining methods (ultrasonographic assessment, microparticle enzyme immunoassay and radioimmunoassay, two-site immunometric assay, chemiluminescence immunoassay) were utilized in 4 separate studies. A total of 7 studies had statistically significant differences between the case and control groups, while the remaining 14 studies exhibited no statistically significant differences (**Table 1**).

### β-hCG MoM Expression Levels and PE

**Figure 2:**
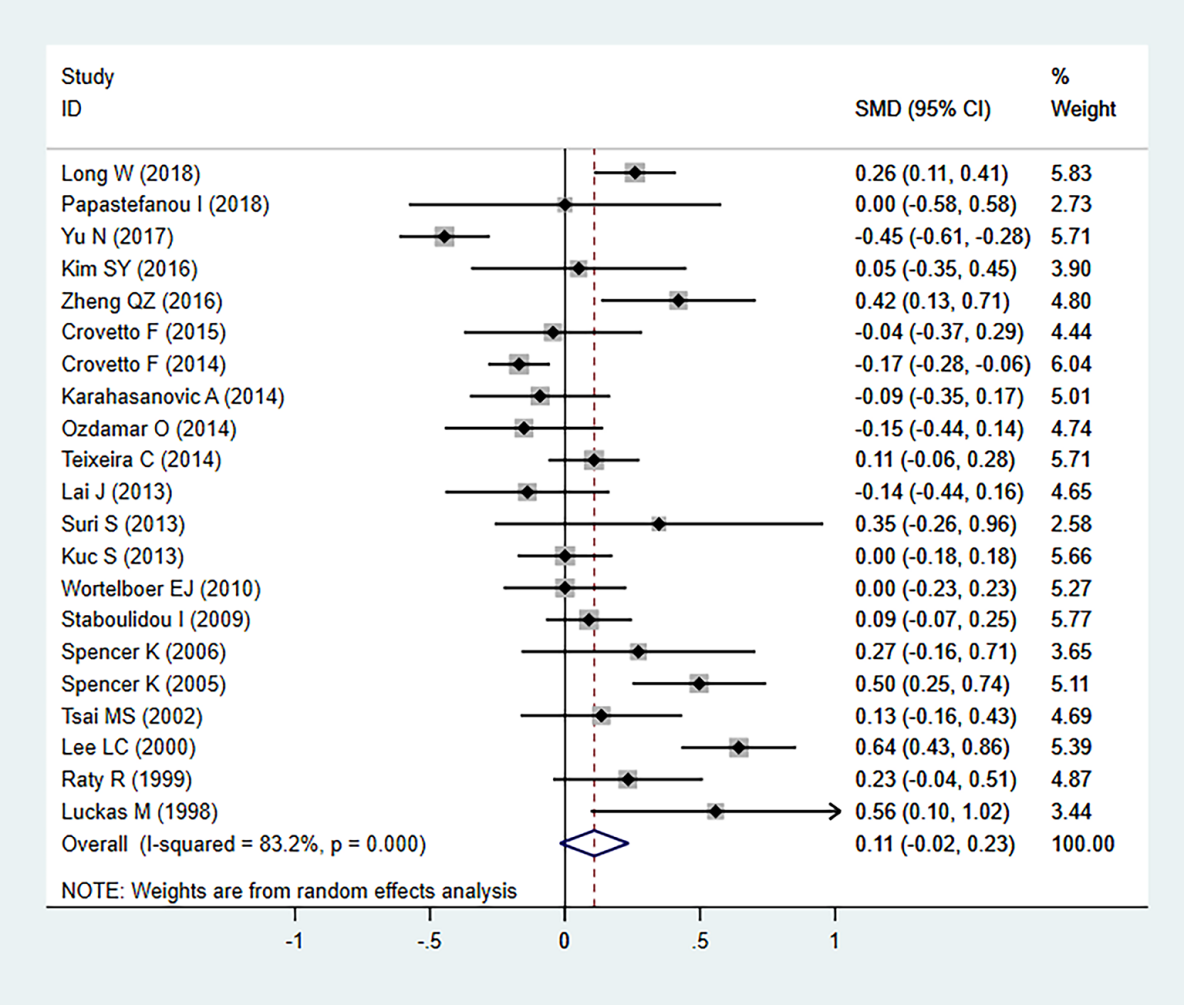
Random-effect model forest plot for the association between serum β-hCG MoM levels and PE.

In our meta-analysis, we detected significant heterogeneity (*I* ^2^ = 83.2%, *P*=000), and therefore the random-effects model was applied. Statistical analysis revealed no significant differences in serum β-hCG MoM levels between patients with PE and controls (SMD=0.108, 95% CI=-0.019-0.235, z=1.67, *P*=0.094) (**Figure 2**). As heterogeneity was observed, the association between the serum β-hCG MoM levels and PE was evaluated for subgroups by sample size, detection period, ethnicity, and diagnostic criteria for PE. Our results demonstrated that, apart from the detection period subgroup, all other subgroups exhibited no significant differences.

**Figure 3:**
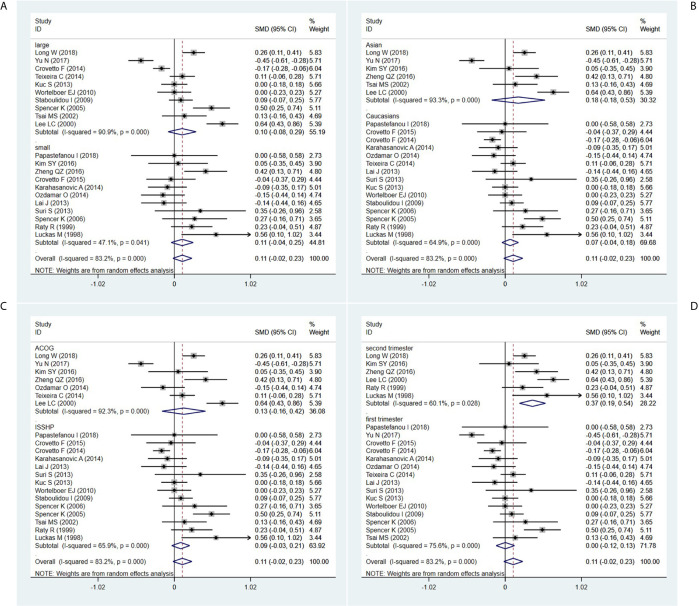
Random-effect forest plot for the subgroup analysis according to **(A)** sample size, **(B)** ethnicity, **(C)** diagnostic criteria for PE, **(D)** detection period.

In the sample size-stratified subgroup analysis, serum β-hCG MoM levels were not significantly different between PE patients in the small size subgroup (SMD=0.106, 95% CI=-0.042-0.253, z=1.40, *P*=0.161) and the large size subgroup (SMD=0.104, 95% CI=-0.082-0.291, z=1.10, *P*=0.273) (**Figure 3A**).

In the ethnicity-stratified subgroup analysis, serum β-hCG MoM levels were not significantly different between PE patients in the Caucasian subgroup (SMD=0.067, 95% CI=-0.043-0.177, z=1.20, *P*=0.230) and the Asian subgroup (SMD=0.176, 95% CI=-0.180-0.532, z=0.97, *P*=0.332) (**Figure 3B**).

In the diagnostic criteria for PE-stratified subgroup analysis, there was no significant difference in serum β-hCG MoM levels between PE patients in the ACOG standard subgroup (SMD=0.126, 95% CI=-0.164-0.417, z=0.85, *P*=0.395) and the ISSHP standard subgroup (SMD=0.086, 95% CI=-0.034-0.206, z=1.40, *P*=0.161) (**Figure 3C**).

In the detection period-stratified subgroup analysis, serum β-hCG MoM levels were higher in PE patients in the second-trimester subgroup (SMD=0.365, 95% CI=0.192-0.539, z=4.12, *P*=0.000) compared with those in the first-trimester subgroup (SMD=0.002, 95% CI=-0.121-0.126, z=0.03, *P*=0.973) (**Figure 3D**).

**Figure 4:**
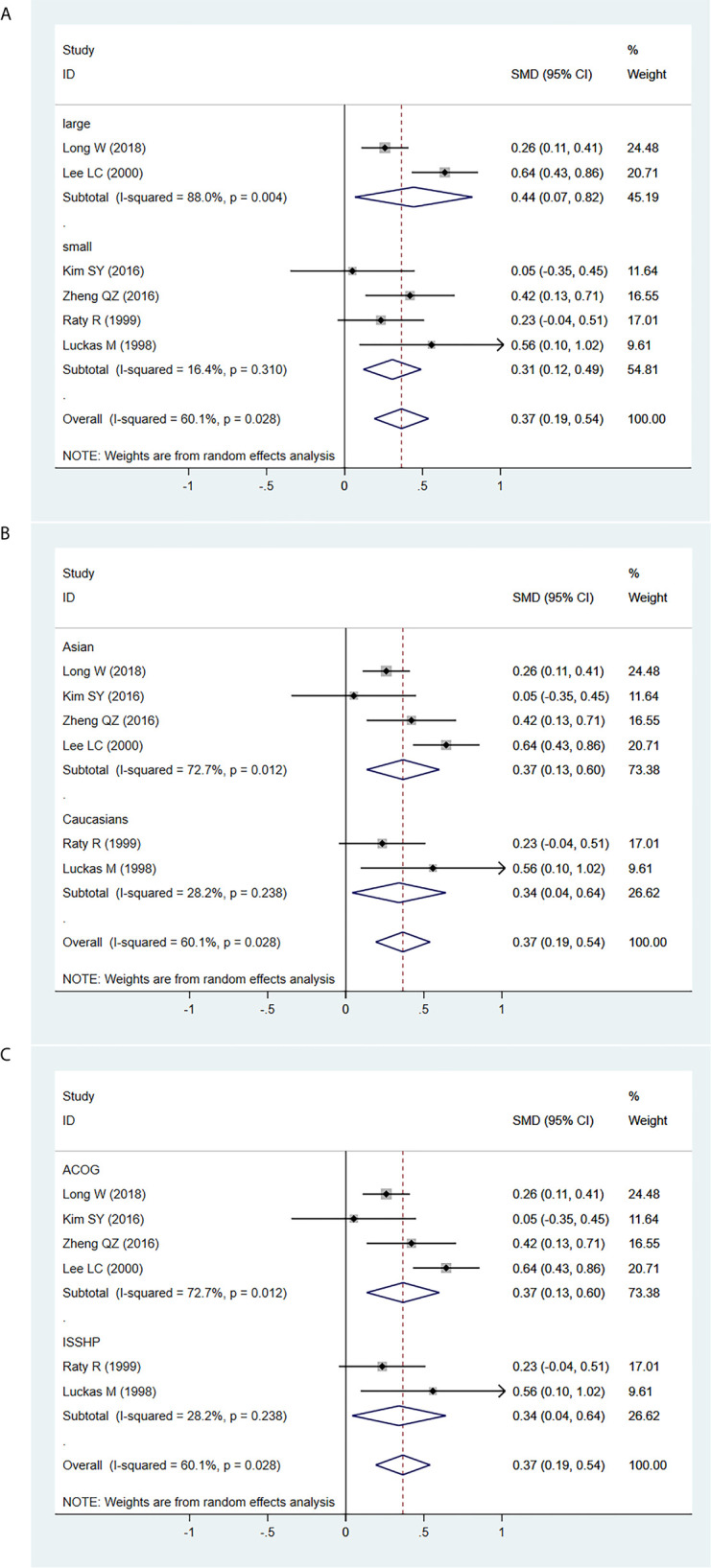
Random-effect forest plot for the subgroup analysis for the association between serum β-hCG MoM levels in the early second trimester and PE according to **(A)** sample size, **(B)** ethnicity, **(C)** diagnostic criteria for PE.

Furthermore, the MoM of serum β-hCG levels in second trimester was significantly higher in PE patients in both the large (SMD=0.444, 95% CI=0.067-0.820, z=2.31, *P*=0.021) and small (SMD=0.307, 95% CI=0.122-0.491, z=3.25, *P*=0.001) sample size subgroups (**Figure 4A**), in both the Asian (SMD=0.367, 95% CI=0.135-0.599, z=3.10, *P*=0.002) and Caucasian (SMD=0.341, 95% CI=0.041-0.640, z=2.232, *P*=0.026) populations (**Figure 4B**), and in both the ACOG diagnostic criteria for PE (SMD=0.367, 95% CI=0.135-0.599, z=3.10, *P*=0.002) and ISSHP diagnostic criteria for PE (SMD=0.341, 95% CI=0.041-0.640, z=2.232, *P*=0.026) subgroups (**Figure 4C**).

**Figure 5:**
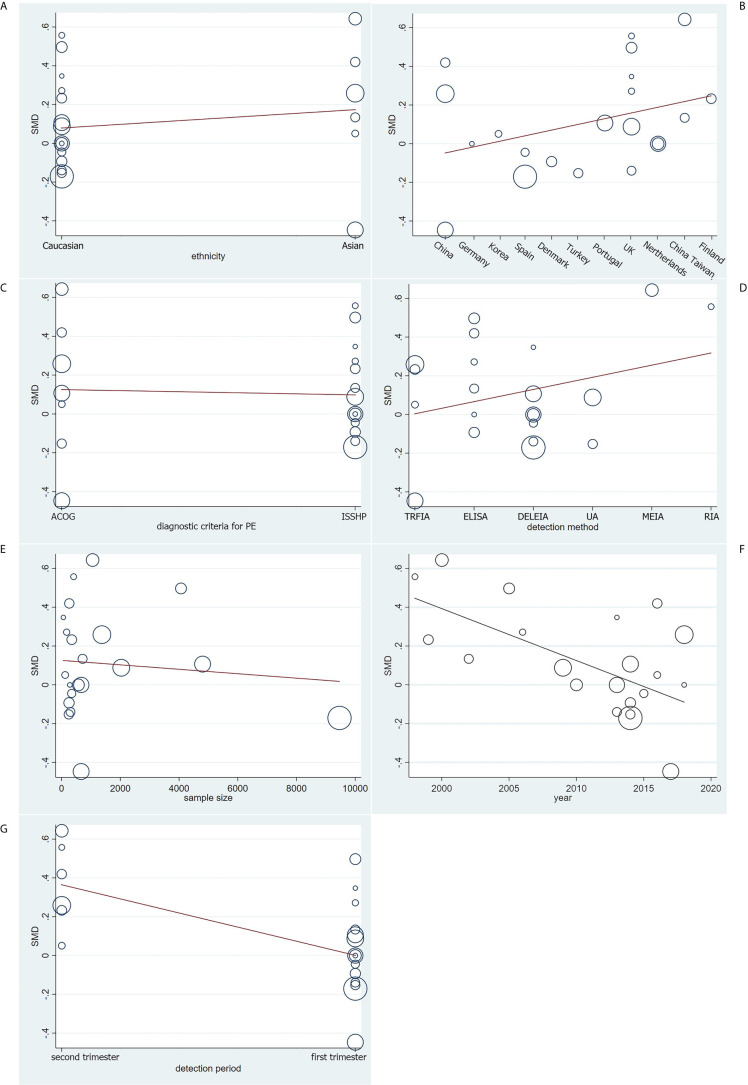
Meta-regression analysis on **(A)** ethnicity, **(B)** country, **(C)** diagnostic criteria for PE, **(D)** detection method, **(E)** sample size, **(F)** year of publication and **(G)** detection period on the basis of the 21 included case-control.

Based on univariate and multivariate meta-regression analyses, ethnicity, country, diagnostic criteria for PE, detection method and sample size were neither the sources of heterogeneity nor the key factors of overall effect size (all *P*>0.05) (**Figure 5** and **Table 2**). However, year of publication (*P*=0.006) and detection period (*P*=0.005) may be considered as sources of heterogeneity.

**Table 2:** Meta-regression analysis of potential source of heterogeneity.

Heterogeneity factors	Coefficient	SE	t	*P* value (adjusted)	95%CI	
					LL	UL
Year of publication	-0.027	0.009	-3.09	0.006	-0.045	-0.009
Ethnicity	0.095	0.137	0.69	0.499	-0.193	0.382
Country	0.030	0.019	1.57	0.133	-0.010	0.069
Diagnostic criteria for PE	-0.027	0.133	-0.21	0.839	-0.310	0.251
Detection method	0.063	0.049	1.28	0.216	-0.040	0.166
Detection period	-0.364	0.115	-3.16	0.005	-0.605	-0.123
Sample size	-0.000	0.000	-0.43	0.674	-0.000	0.000

LL, Lower limit; UL, upper limit.

### Sensitivity Analysis and Publication Bias

**Figure 6:**
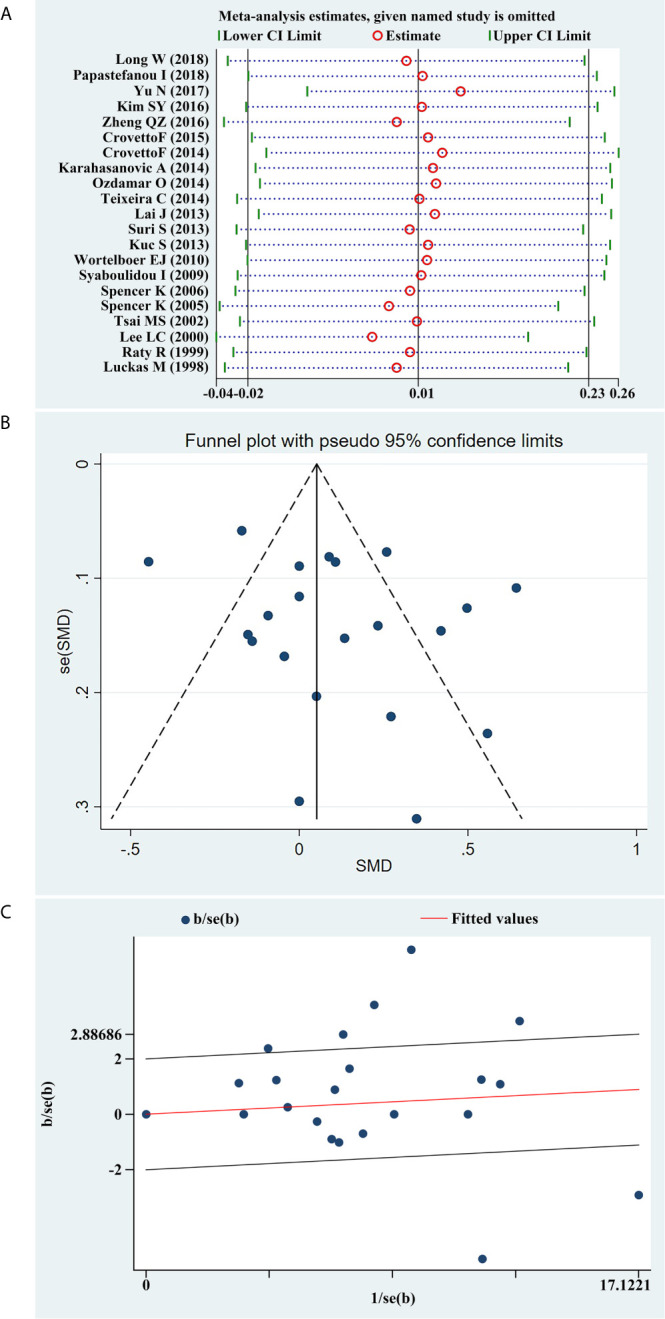
**(A)** Sensitivity analysis of the summary odds ratio coefficients on the difference of free β-hCG MoM values between PE and control subjects. **(B)** Funnel plot of publication biases on the difference of free β-hCG MoM values between PE and control subjects. **(C)** Galbraith plot of publication biases on the difference of free β-hCG MoM values between PE and control subjects.

We conducted a sensitivity analysis of the present meta-analysis, and the results demonstrated that no single study had an impact on the overall estimate of the association between serum β-hCG MoM levels and PE progression (**Figure 6A**). In addition, Begg’s funnel plot (**Figure 6B**) and Galbraith plot (**Figure 6C**) did not reveal any obvious asymmetry, and Egger’s regression test suggested the absence of publication bias (*P*=0.173).

## Discussion

β-hCG is a glycoprotein hormone synthesized by placental trophoblast cells, and hCG stimulates trophoblast proliferation and invasion, thereby promoting placental growth (46). The abnormal increase in hCG levels is considered to be the result of decreased placental perfusion related to low oxidation stemming. The histological studies conducted by Lieppman et al. demonstrated that cytotrophoblasts’ abnormal placentation was induced by hypoxia (47). Some studies have reported an association between β-hCG and PE, but their results were different; for example, the serum β-hCG concentrations or β-hCG MoM levels were significantly higher in pregnancies that subsequently developed PE (48). A higher β-hCG MoM (≥2.31) was associated with a higher risk of PE (15). High levels of β-hCG MoM (≥95% percentile) were associated with severe PE (RR 2.5-11.7) (49). Using a cutoff concentration of 2.0 MoM of β-hCG in both the primigravida and multigravida groups, the area below the curve, sensitivity, specificity, positive predictive value, negative predictive value and positive likelihood ratio were 0.96 and 0.95, 88.5 and 100%, 92.0 and 85.6%, 0.46 and 0.25, 0.99 and 1.0, and 11.1 and 6.9 for the two groups, respectively (50). Other studies suggested that the serum β-hCG level is increased in pregnancies with established PE and in the third trimester before clinical onset of the disease, but is reduced or unaltered at 11-13 weeks of gestation (12, 35, 51–54). In 2015, a meta-analysis demonstrated that the serum β-hCG MoM levels were significantly increased in PE patients compared with those in the control group, and screening for serum β-hCG MoM levels may be useful for the early identification of pregnancies who are at risk of developing PE (9). However, most recent studies suggested that the serum β-hCG MoM levels were not significantly different between patients with PE and normal pregnancies (15–18). Therefore, the predicted value of serum β-hCG MoM levels for PE must be re-evaluated.

The MoM of β-hCG is a concentration index representing the MoM of normal β-hCG levels found in healthy pregnant women (55). We used a meta-analysis-based approach to perform an assessment of the predictive value of β-hCG MoM in the early detection of PE. The results demonstrated that the serum β-hCG MoM levels of PE patients were not significantly different compared to those of healthy women in the first trimester, but may have a certain clinical value for predicting PE in the early second trimester, which is consistent with the results of previous studies (13, 14, 36, 50, 56). Gestational week may affect the correlation between β-hCG MoM and PE. Serum β-hCG MoM levels improve the prediction of PE in the early second trimester, but the improvement is small, and it may not be a useful marker of PE in the first trimester. Therefore, it may be necessary to combine other indicators to improve the sensitivity and specificity of predicting PE. In order to identify the influence of other factors, such as ethnicity, sample size and diagnostic criteria for PE, on the predictive value of β-hCG MoM in PE patients, a subgroup analysis was performed. Regardless of ethnicity-stratified analysis or sample size-stratified analysis or diagnostic criteria for PE-stratified analysis, we found that β-hCG was of no value for the prediction of PE in 18 included studies. However, for the 6 studies in which detection was only performed in the second trimester, the levels of serum β-hCG MoM were significantly higher in patients with PE in both Asian and Caucasian populations.

Although this was a meta-analysis on the association between β-hCG MoM and PE, the whole sample size was small and no subgroup analysis was performed for detection period. A number of scholars have repeatedly investigated the value of β-hCG for PE in recent years, but their results differ, and the majority indicate that there is no association between β-hCG MoM and PE; thus, it was deemed necessary to perform another meta-analysis. We hypothesized that the reason for these results maybe that the pathological changes of PE in the first trimester are mainly caused by defective trophoblast invasion, leading to reduced uteroplacental blood flow at approximately 12 weeks of gestation, with little or no influence of β-hCG levels, whereas subsequent oxidative stress and other reactions occurring in the placenta in the early second trimester induce higher β-hCG levels (57).

There were certain limitations to the present study. First, the heterogeneity between studies was high. To overcome this, various subgroup analyses were performed to explore the different causes of heterogeneity, thus yielding better accuracy values in the subgroup analyses. We did not perform meta-regression on the early second trimester subgroup to find its heterogeneity source due to the number of included studies being <10. Second, the treatment was not randomized in the study, which means the predictive value for β-hCG MoM levels from this sample set could not be identified. Third, the methods used for measuring β-hCG MoM levels were not the same, and the 6 different methods may cause selection bias. Fourth, three of the included articles were not high-quality articles, which may have affected the final results. Fifth, considering the relatively high morbidity and mortality rates associated with PE, it would be best to identify as many patients as possible; higher-risk populations maybe excluded due to the narrower false-positive rate, and the lack of detection of false-positive and false-negative rates may have limited the reliability of our results. Finally, the detection period of β-hCG was only divided into two stages instead of some definitive gestational weeks. Perhaps a more narrowed detection period could further enhance the predictive value of serum β-hCG MoM level.

## Conclusion

In conclusion, it was demonstrated that serum β-hCG MoM levels were higher in PE patients in the early second trimester compared with those in healthy pregnant women, which may represent a possible screening method for early prediction and potential interventions in PE. Further investigations are required, including a prospective assessment of patients with PE and evaluation of the optimal cut-off value for β-hCG MoM, in order to develop an applicable predictive tool for routine pregnancy monitoring and management, maximize the predictive potential sensitivity and specificity, and elucidate the potential underlying mechanism.

## Data Availability Statement

The original contributions presented in the study are included in the article. Further inquiries can be directed to the corresponding authors.

## Author Contributions

XZ and ZH have worked on the whole article, who contributed equally to this article. FS and ZX were corresponding authors who have guided the research. All authors agree to be accountable for the content of the work. All authors contributed to the article and approved the submitted version.

## Funding

This study was supported by Tibet Local Science and Technology Project guided by Central Government (Grant No. XZ202001YD0005C), Scientifical Funds of Medical Assistance Program for Tibet from Tibet Health Committee(Grant No. XZ2020ZR-ZY78(Z)), the Natural Science Funds of Liaoning (Grant No.: 2019-BS-073).

## Conflict of Interest

The authors declare that the research was conducted in the absence of any commercial or financial relationships that could be construed as a potential conflict of interest.
